# Comparing the Effects of Epley Maneuver and Cinnarizine on Benign Positional Paroxysmal Vertigo; A Randomized Clinical Trial

**DOI:** 10.31661/gmj.v8i0.866

**Published:** 2019-01-01

**Authors:** Masoumeh Saeedi, Mohammad Hossein Khosravi, Mohammad Ehsan Bayatpoor

**Affiliations:** ^1^Department of Otorhinolaryngology-Head and Neck Surgery, Faculty of Medicine, Baqiyatallah University of Medical Sciences, Tehran, Iran; ^2^Student Research Committee, Baqiyatallah University of medical sciences, Tehran, Iran; ^3^International Otorhinolaryngology Research Association (IORA), Universal Scientific Education and Research Network (USERN), Tehran, Iran

**Keywords:** Benign Paroxysmal Positional Vertigo, Cinnarizine, Epley Maneuver, Vertigo

## Abstract

**Background:**

The fastest and safest treatment method of BPPV is repositioning maneuvers. In Iran, this methods are not widely used, and many physicians use medical therapy, despite their side effects, for management of BPPV.

**Materials and Methods:**

In this randomized clinical trial patients with BPPV were randomly allocated to Epley repositioning maneuver or Cinnarizine (25mg every 8 hours) for two weeks. The patients were evaluated for symptoms using visual analogue scale (VAS) scoring system before intervention, first and second weeks after intervention. In the second and third visitd the results of hallpike test was recorded for both groups.

**Results:**

43 patients with a mean age of 46.88±11.08 years in two Epley and Cinnarizine group underwent analysis. The mean VAS score for improvement of symptoms after intervention was 1.66±1.06 in Epley and 1.50±0.91 in Cinnarizine group (P=0.57).

**Conclusion:**

we found that there is no significant difference between Epley maneuver and Cinnarizine for treatment and controlling symptoms of BPPV.

## Introduction

Benign positional paroxysmal vertigo (BPPV) is one of the most important causes of vertigo with a prevalence of 11-64 per 100000 people and a life- long prevalence of 2.4% [1]. It is considered that BPPV symptoms occur when otoconia are dislodged from otolith structures and attached to cupula in semicircular canals [2-4]. It is believed that posterior semicircular canal is the common site of involvement; however horizontal and superior canals are rarely affected [1]. BPPV is routinely managed by drugs such as Cinnarizine, an histamine antagonist and calcium channel blocker, which improves vertigo through effecting on calcium channels in peripheral vestibular labyrinth [5, 6]. Repositioning maneuvers are the fastest and safest non-surgical practical treatment of BPPV that are performed by changing head and body positions. These treatments are completely available and take only 5 minutes [7]. Epley, a specific maneuver for posterior semicircular canal BPPV with a high rate of success (85-90%), is one of these maneuvers which has no sever complications except nausea and vomiting [8-11]. According to previous studies, improvement rate of symptoms ranges between 50 to 95% one to two weeks after treatment [12-14]. Applying this therapeutic method is not common in Iran and most of physicians prescribe medications for patients with vertigo. According to this issue and that few studies have been yet conducted to compare these two methods, we aimed to compare the effectiveness of Epley maneuver and Cinnarizine in treatment of BPPV.

## Materials and methods

**Figure 1:**
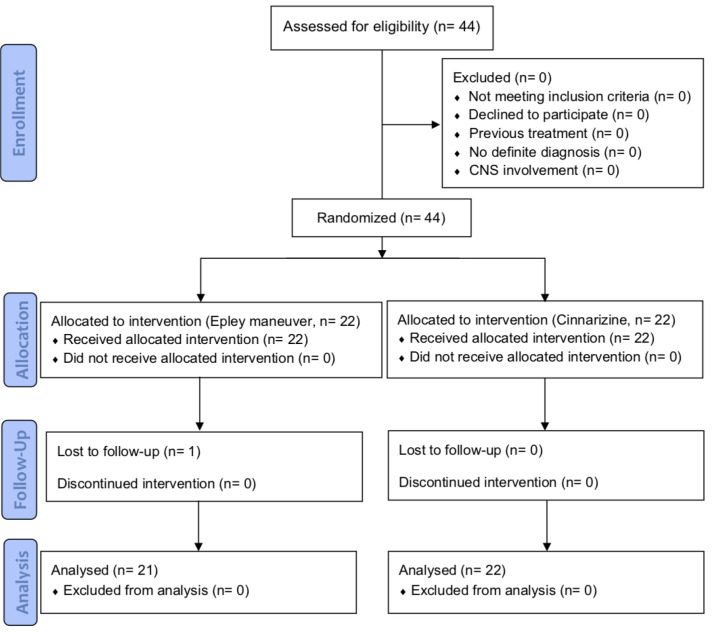
Study flowchart

This randomized clinical trial was registered at ethics committee of Baqiyatallah University of Medical Sciences (Reference no: IR.BMSU.REC.1391.112) and Iranian Registry of Clinical Trials (Reference no: IRCT2016101717413N19). Figure-1 shows a flowchart of the trial. Patients with Benign Positional Paroxysmal Vertigo (BPPV) attending to vertigo clinic of Baqiyatallah hospital, Tehran, Iran in 2014 and 2015 were assessed for eligibility. All patients were informed of study process and possible side effects. A written informed consent was obtained from all of patients. Patients with definite diagnosis of BPPV, up to 2 days after diagnosis, and those who have not received any treatments for BPPV were included. BPPV diagnosis was made by single physician based on history taking and hallpike test. Patients with no definite diagnosis and those suspicious for other causes of vertigo, especially CNS involvement, were excluded from the study. Patients were randomized to two groups using random number table: the first group (A) underwent Epley repositioning maneuver by single physician and the second group (B) used Cinnarizine (25mg every 8 hours) for two weeks. The block randomization was accomplished by the clinic’s automatic turn system and patients were randomized into 2 groups at a ratio of 1:1 and a block size of 4. In the first visit, results of hallpike test was recorded for both groups and after first Epley maneuver, group A patients were recommended to place their head 45 degrees upper the body level during sleep for two days. The patients were evaluated for symptoms using visual analogue scale (VAS) scoring system before intervention, first and second weeks after intervention. In the second and third visit, Epley maneuver was repeated in group A patients and the results of hallpike test was recorded for both groups. Data were analyzed using SPSS software version 21 (SPSS Inc., Chicago, IL) for Microsoft Windows. Sample size was calculated using

“N= 2(Z _1-α_ + Z _1-β_) ^2^ * P(1-P) / ( P0-P1) ^2^ “

formula. The chi square test was used to compare categorical variables in the 2 groups. Independent sample *t* test and its nonparametric equivalent were used to compare the values before and after treatment within the groups. A P value of less than 0.05 was considered as statistically significant.

## Results

Eventually 43 patients (25 male and 18 female) with a mean age of 46.88±11.08 years in two Epley (48.19±9.82) and Cinnarizine group (45.63±12.26) underwent analysis (P=0.457). Epley and Cinnarizine groups consisted 13 and 12 male patients, respectively (P=0.42). In Epley group 17 (80%) patients and in Cinnarizine group 9 (40%) patients complained of disequilibrium (P=0.012). Eight (38%) patients in Epley group and 6 (27%) patients in Cinnarizine group had head-lightness (P=0.44). Most of patients in Epley (76%) and Cinnarizine (86%) groups had nausea (P=0.232); while 6(28%) patients in Epley group and 2 (9%) patients in Cinnarizine group had vomiting during vertigo (P=0.175). In Epley group 7(33%) and in Cinnarizine group 3 (13%) patients mentioned sweating during vertigo (P=0.062). Symptoms were present for less than one month in 12(57%), one month to one year in 8(38%) and more than one year in 1(4%) patients in Epley group. In Cinnarizine group 9(40%), 10(45%) and 3(14%) patients had symptoms for less than month, one month to one year and more than one year, respectively (P=0.433). Changing position was mentioned as provoking agent by 14(66%) patients in Epley and 10(45%) patients in Cinnarizine group. Valsalva maneuver worsened symptoms in 4(18%) patients in Cinnarizine group. In 7(33%) patients in Epley group and 8(36%) in Cinnarizine group symptoms were worsened spontaneously (P=0.095). Four patients (19%) in Epley and 8(36%) in Cinnarizine group had a history of head trauma (P=0.206). In Epley group 9(42.9%) patients and in Cinnarizine group 14(63.6%) patients had a positive history for other ear diseases (P=0.172). Table-1 summarizes other complaints of patients prior to intervention. The mean VAS score for improvement of symptoms after intervention was 1.66±1.06 in Epley and 1.50±0.91 in Cinnarizine group (p=0.57). Table-2 shows improvement of symptoms reported by patients. In Epley group 3(14%) patients and 1(4%) in Cinnarizine group reported “complete resolution of symptoms”. While 1(4.8%) patient in Epley and 1(4.5%) patient in Cinnarizine group reported “no changes” in symptoms (P=0.34). There was no statistically significant relation between mean VAS score (1.58±0.981) and mean age of patients based on Pearson Correlation (P=0.449). The mean VAS score of female patients was 1 unit more than male patients (P=0.001, Table-3). Patients with longer disease duration, spontaneous exacerbation of symptoms, all three ototonic symptoms (sweating, nausea and vomiting), no history of head trauma or other inner ear disease and those without otalgia had a significantly higher VAS score (p<0.05, Table-3). Mean VAS score was not significantly different between patients with or without hearing loss (P=0.336).

## Discussion

We found that there is no significant difference between Epley maneuver (A) and Cinnarizine (B) for management of symptoms in patients with BPPV. All demographic characteristics and symptoms except disequilibrium were not significantly different between two groups before intervention. Also there was no statistically significant difference between A and B groups for mean VAS score. Female patients and those with longer disease duration, spontaneous exacerbation of symptoms and all three ototonic symptoms (sweating, nausea and vomiting) had a significantly higher VAS score in both groups. While this was significantly lower in patients with history of head trauma or other inner ear disease and otalgia.

Our findings confirm the results of Cohen *et al*. study for effect of Epley maneuver with sleep position recommendations in controlling symptoms of patients with BPPV and also Steenerson *et al*. study for effect of repositioning maneuvers on the treatment of BPPV [15, 16]. The present study has considered limitations mentioned in Helminski *et al*. study and assessed confounders like history of head trauma and other inner ear disease in the patients [17]. Sundararajan *et al*. evaluated the adjunctive effect of labyrinth sedatives to Epley maneuver on the treatment of BPPV; while we compared medical treatment with Epley maneuver in two groups of patients. In Sundararajan *et al*. study patients underwent one week of treatment and were followed for four weeks. The results are in concordance with the present study in effect of Epley maneuver on eliminating symptoms of patients. In contrast with the present study, they concluded that labyrinth sedatives were not effective which may be a result of different study designs [18]. Evaluating the effect of Epley maneuver, with and without Betahistine, on the treatment of BPPV, Guneri *et al.* concluded that medical treatment is effective as well as repositioning maneuver which is in agreement with the present study [19]. In accordance with the present study, Foster *et al.* confirmed the effect of Epley maneuver on the treatment of BPPV. They have also presented “Half somersault” exercise as a more resistant maneuver than Epley with less complications. The six-month follow up time has made their study distinguished [20]. Maslovara *et al*. study is in agreement with the present study for the effect of Epley maneuver on BPPV. They have mentioned neck brace, neck movement limitation and upright sleep position associated with Epley maneuver are accompanied with less relapses than Epley maneuver alone. Maslovara *et al*. have also reported notable effects of Mastoid vibration techniques [21]. Kim* et al*. have reported inner ear disease and hearing loss in the same side as factors related to treatment failure with Epley manuever which is in concordance with our study [22]. In a similar study Sato *et al*. have mentioned head trauma as a risk factor for BPPV treatment failure with Epley maneuver; while inner ear diseases had not this condition [23]. Evaluating the effect of Semont maneuver as an alternative method for repeating Epley maneuver, Oh *et al*. reported that there is no superiority for either of the maneuvers in treatment of patients with posterior canal benign paroxysmal positional vertigo [24]. In another study Kaur *et al*. evaluated BPPV patients in three Epley maneuver alone, Betahistine alone and Betahistine plus Epley maneuver groups. They concluded that concurrent prescription of Betahistine and Epley maneuver is superior to other two options. They have also suggested Betahistine alone as an appropriate alternative treatment for patients who cannot tolerate repositioning maneuvers [25]. The present study had some limitations. Blinding was not available in the present study because the two interventions were completely different. A relatively low sample size is another limitation of the present study.

## Conclusion

In conclusion we found that there is no significant difference between Epley maneuver and Cinnarizine for treatment and controlling symptoms of BPPV. So we suggest Epley maneuver for management of BPPV because of its lower expenses in comparison with medication. In addition this could be more effective in elderly patients, especially those with various prescribed medications, to decrease the complications and quantity of drugs. Also further studies are suggested with more follow up period and assessment of quality of life of patients using standard questionnaires. It is recommended that patients be evaluated in more groups for evaluating the effects of different drugs, with or without repositioning maneuvers.

## Conflict of interest

There are no conflicts of interest in terms of the present study.

**Table 1:** Other Complaints of Patients Prior to Intervention

	**Epley maneuver** ** N=21**	**Cinnarizine** **N=22**	**Total** **N=43**	**P-value** ^*^
**Age (Mean±SD)**	48.19±9.82	45.63±12.26	46.88±11.08	0.45
**Otalgia**	2(9.5%)	4(18.2%)	6(14%)	0.66
**Tinnitus**	12(57.1%)	13(59.1%)	25(58.1%)	0.89
**Increased Discharge**	9(42.8%)	13(59.1%)	22(51.1%)	0.42
**Hearing loss**	9(42.8%)	8(36.4%)	17(39.5%)	0.63

**Table 2:** Changes of Symptoms Reported by Patients

	**Complete Resolution**	**Significantly Improved**	**Improved**	**Slightly Improved**	**No change**
**Epley group** **N=21**	3(14.3%)	6(28.6%)	8(38.1%)	3(14.3%)	1(4.8%)
**Cinnarizine group** **N=22**	1(4.5%)	13(59.1%)	5(22.7%)	2(9.1%)	1(4.5%)
**Total** **N=43**	4(9.3%)	19(44.2%)	13(30.2%)	5(11.6%)	2(4.7%)
**P-value**			0.34		

**Table 3:** Relation between Mean VAS Score and Patients Characteristics after Intervention

		**Mean VAS score (±SD)**	**P-value**
**Gender**	Male	1.16±0.62	0.001
	Female	2.16±1.09	
**Disease duration **	<1 month	0.95±0.58	
	>1month, <1 year	1.88±0.67	0.001
	> 1 year	3.5±0.57	
**Provoking agent **	Changing position	1.04±0.62	
	Valsalva maneuver	1	0.001
	Spontaneous	2.60±0.73	
**Ototonic symptoms**	Nausea (N=27)	1±0.55	
	Sweating (N=8)	2.25±0.46	0.001
	Nausea and Vomiting (N=6)	2.83±0.75	
	Sweat and Nausea and Vomiting (N=2)	3±1.41	
**History of Head Trauma**	Yes (N=12)	0.66±0.49	0.01
	No (N=31)	1.93±0.89	
**History of inner ear disease**	Yes (N=23)	0.82±0.38	0.02
	No (N=20)	2.04±0.68	
**Otalgia**	Yes (N=6)	0.66±0.51	0.012
	No (N=37)	1.72±0.96	
**Hearing loss **	Yes (N=17)	1.41±0.71	0.336
	No (N=26)	1.69±1.12
